# Caffeine prevents kidney stone formation by translocation of apical surface annexin A1 crystal-binding protein into cytoplasm: *In vitro* evidence

**DOI:** 10.1038/srep38536

**Published:** 2016-12-07

**Authors:** Paleerath Peerapen, Visith Thongboonkerd

**Affiliations:** 1 Medical Proteomics Unit, Office for Research and Development, Faculty of Medicine Siriraj Hospital, and Center for Research in Complex Systems Science, Mahidol University, Bangkok, Thailand

## Abstract

Recent large 3 cohorts have shown that caffeinated beverage consumption was associated with lower risk of kidney stone disease. However, its protective mechanisms remained unknown and had not been previously investigated. We thus evaluated protective effects of caffeine (1 μM–10 mM) on calcium oxalate monohydrate (COM) kidney stone formation, using crystallization, crystal growth, cell-crystal adhesion, Western blotting, and immunofluorescence assays. The results showed that caffeine reduced crystal number but, on the other hand, increased crystal size, resulting in unchanged crystal mass, consistent with crystal growth that was not affected by caffeine. However, caffeine significantly decreased crystal-binding capacity of MDCK renal tubular cells in a dose-dependent manner. Western blotting and immunofluorescence study of COM crystal-binding proteins revealed significantly decreased level of annexin A1 on apical surface and its translocation into cytoplasm of the caffeine-treated cells, but no significant changes in other COM crystal-binding proteins (annexin A2, α-enolase, HSP70, and HSP90) were observed. Moreover, caffeine decreased intracellular [Ca^2+^] but increased [Ca^2+^] secretory index. Taken together, our findings showed an *in vitro* evidence of the protective mechanism of caffeine against kidney stone formation via translocation of annexin A1 from apical surface into cytoplasm to reduce the crystal-binding capacity of renal tubular epithelial cells.

Nephrolithiasis or kidney stone disease is caused by deposition of mineralized crystals, which later become stone(s), within the kidney. The incidence of this disease remains considerably high around the globe^1^. Calcium oxalate monohydrate (COM) is the most common mineralized crystal found in the stone matrix^2^. There are several approaches to remove kidney stones from stone patients, e.g. shock wave lithotripsy and surgery^3^. However, 50% of the stone formers have recurrent stones within 10 years after the stone removal^4^,^5^. The high recurrence rate together with the remaining high or even increasing incidence of new cases of kidney stone disease indicates that prevention of this disease in the past (e.g. hydration, alkali treatment) was unsuccessful. Hence, recent stone research has focused on defining protective strategies to prevent new and recurrent kidney stone formation. One among those strategies is to directly modulate COM crystallization, crystal growth, crystal aggregation, and crystal adhesion onto renal tubular epithelial cells^6^,^7^,^8^.

Interestingly, recent large 3 cohorts, including The Health Professionals Follow-Up Study (HPFS) cohort, The Nurses’ Health Study (NHS) I cohort, and The Second NHS (NHS II) cohort with a total of >200,000 participants, have found that consumption of caffeinated beverages was associated with 26–31% lower risk of kidney stone disease^9^. In addition, decaffeinated coffee, which contained much lower amount of caffeine in the coffee (up to 8.4% of the caffeinated coffee)^10^, could also reduce the incidence of kidney stone disease but with a lower degree (by 16%)^11^. As caffeine is commonly and daily consumed worldwide, it may be the preventive strategy of choice to reduce new and recurrent kidney stone incidence in the future. Although the strong association between caffeine intake and reduction of kidney stone incidence has been found, protective mechanisms of caffeine against kidney stone disease remained unknown and had not been previously investigated.

Recently, several studies have shown significant roles of COM crystal-binding proteins (e.g. annexin A1, α-enolase, heat shock protein 90 (HSP90)) on apical membranes of renal tubular epithelial cells as potential crystal receptors essential for COM crystal adhesion on apical surface of the cells, intratubular COM crystal retention, and subsequently, stone formation^7^,^12^,^13^,^14^,^15^. Interestingly, expression of these potential COM crystal receptors could be altered by stone modulators or risk factors. For example, high-calcium state increased surface annexin A1, whereas high-oxalate condition increased surface α-enolase on apical membranes of renal tubular epithelial cells^12^,^13^. Nevertheless, whether caffeine also affected expression levels of these potential COM crystal receptors remained to be elucidated.

The present study thus aimed to evaluate the protective effects of caffeine on COM kidney stone formation at early phases, including crystallization, crystal growth, and cell-crystal adhesion. Because the initial site of kidney stone formation has been hypothesized (and later supported by histopathological evidence) to occur at distal nephron segment, Madin-Darby Canine Kidney (MDCK) cell line that was initially derived from renal cortical tissue and showed many properties of distal renal tubular epithelium was used as an *in vitro* model in this study^16^,^17^. Effects of caffeine on expression levels and localizations of known COM crystal-binding proteins (including annexin A1, annexin A2, α-enolase, HSP70 and HSP90) were also determined by Western blot analysis and immunofluorescence staining followed by laser-scanning confocal microscopy. Moreover, potential mechanisms underlying caffeine-induced changes were examined (i.e. by measuring intracellular [Ca^2+^] and [Ca^2+^] secretory index, as well as determination of effects of high-calcium and low-calcium treatments on expression levels of the affected receptors).

## Results and Discussion

**Figure 1:**
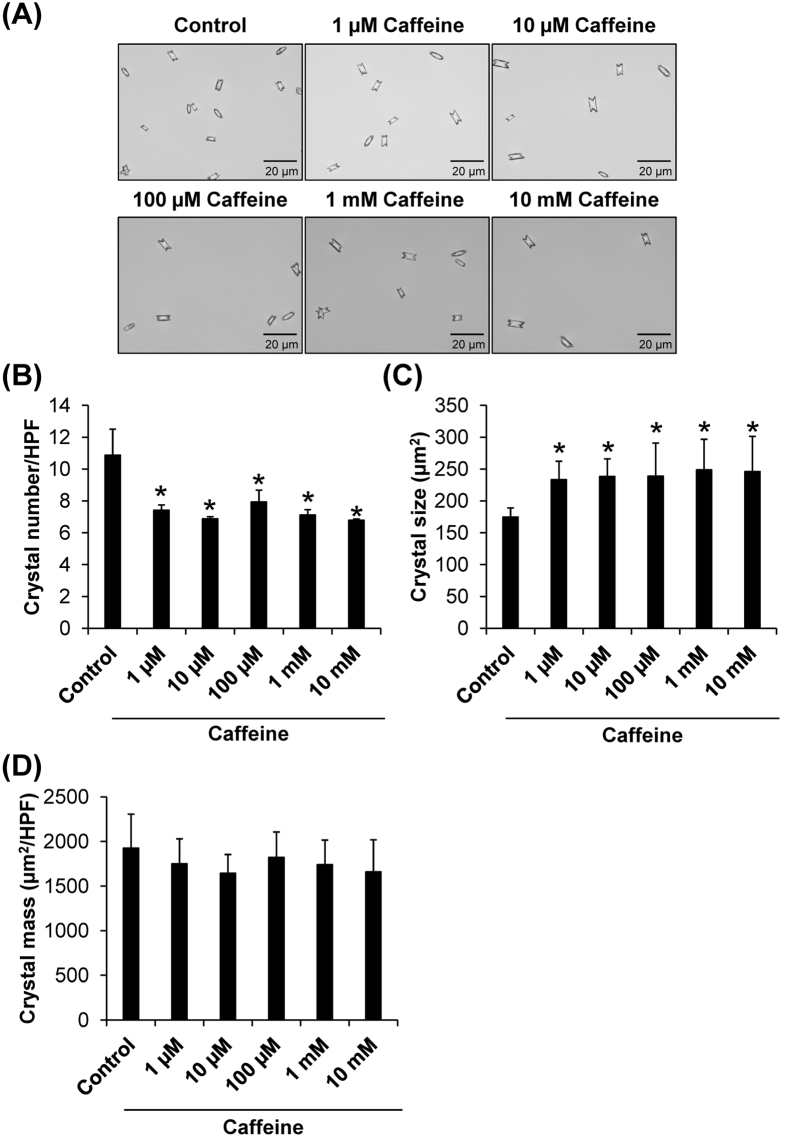
Crystallization assay. (**A**) Morphology of COM crystals obtained after crystallization in the absence or presence of various doses of caffeine. Original magnification was 400× for all panels. (**B**) Crystal number was counted from at least 15 high-power fields (HPFs). (**C**) Crystal size was measured from at least 100 individual crystals in each well. (**D**) Crystal mass was calculated using ***Equation 1***: [*Crystal mass (μm*^2^*/HPF*) = *Average crystal size (μm*^2^) × *Crystal number (/HPF*)]. Each bar represents mean ± SEM from 3 independent experiments. *p < 0.05 vs. control.

The first step of COM kidney stone formation is crystallization, which is a transition of calcium and oxalate ions from solubilized liquid phase to the solid phase as crystalline particles. Crystallization of supersaturated calcium and oxalate ions in renal tubular fluid depends on several factors including calcium and oxalate concentrations, temperature, pH, fluid flow rate, and other inorganic/organic compounds concomitant in the fluid^18^,^19^. Crystallization assay was performed to evaluate effects of caffeine on COM crystallization. Size and number of the COM crystals in the absence or presence of various doses of caffeine were analyzed. The crystal morphology was observed under an inverted light microscope (Fig. 1A). Quantitative data showed that caffeine significantly reduced number of COM crystals, but on the other hand increased the crystal size, independent of its dosage (Fig. 1B and C). We then evaluated the crystal mass, which is more relevant to evaluate or reflect degree of crystallization. The data demonstrated that crystal mass indeed was not affected by caffeine (Fig. 1D). These findings indicated that caffeine, while affected crystal number and size, did not affect the final product of COM crystallization.

**Figure 2:**
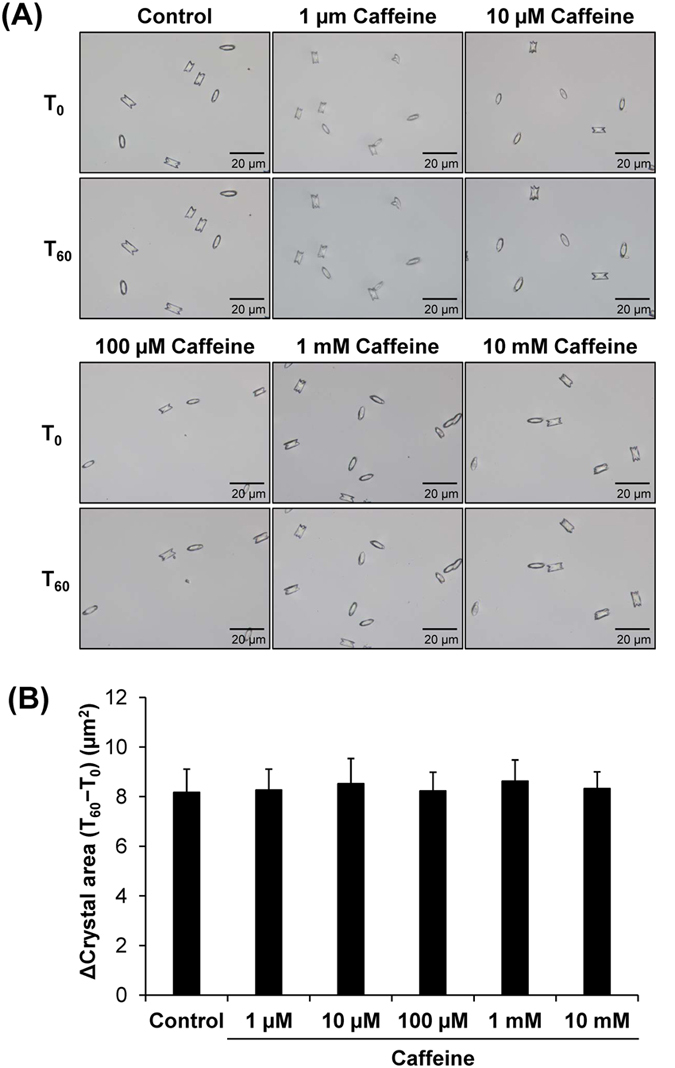
Crystal growth assay. (**A**) Crystal images before (T_0_) and after (T_60_) growing the crystals in the absence or presence of various doses of caffeine for 60 min. Original magnification was 400× for all panels. (**B**) The crystal growth was analyzed and is reported as Δ crystal area using ***Equation 2:*** [*Δ Crystal area (μm*^2^) = *Crystal area at T*_*60*_ (*μm*^2^) − *Crystal area at T*_*0*_(*μm*^2^)]. Each bar represents mean ± SEM from 3 independent experiments.

After crystallization, the crystals can further grow to a bigger size. COM crystal growth assay was performed and the altered crystal area, representing crystal growth, was measured after the crystals were incubated in the absence or presence of various doses of caffeine for 1 h. Similar to crystal mass data in the crystallization assay, the crystal growth assay showed no significant effects by any doses of caffeine (Fig. 2). These results indicated that caffeine had no effect on COM crystal growth. This was not unexpected as caffeine is a purine base in methylxanthine family, which has a very low affinity to bind calcium^20^. Therefore, it is unlikely that caffeine can directly intervene crystal molecular structure that is a critical step required for crystal growth modulatory activity.

**Figure 3:**
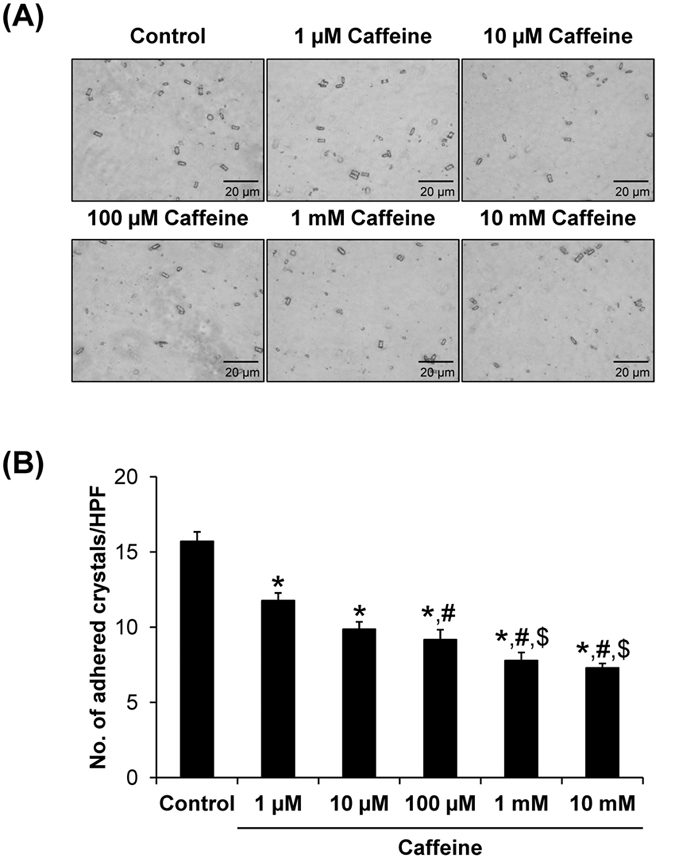
Cell-crystal adhesion assay. (**A**) MDCK cells were pre-incubated with various doses of caffeine for 3 h prior to cell-crystal adhesion assay (the cells pre-incubated with no caffeine served as the control). Original magnification was 400× for all panels. (**B**) The adherent crystals remained on the cell surface after vigorous washes were counted from at least 15 HPFs. Each bar represents mean ± SEM from 3 independent experiments. *p < 0.05 vs. control; ^**#**^*p* < 0.05 vs. 1 μM caffeine; and ^$^*p* < 0.05 vs. 100 μM caffeine.

Crystal adhesion on renal tubular epithelial cell surface is a crucial step for intratubular kidney stone formation. As both crystallization and crystal growth assays showed no significant modulatory effects of caffeine on the crystals directly, we then performed cell-crystal adhesion assay to address whether caffeine affected this critical step of kidney stone formation. Note that caffeine was incubated with the cells for 3 h prior to cell-crystal adhesion assay, whereas its direct incubation with COM crystals in crystallization and crystal growth assays was only 1 h. The longer duration of incubation with the cells was designed because cellular response to caffeine (or any other) treatment (particularly, change in expression levels and/or localizations of potential receptors) usually takes time, whereas direct effects on crystallization and crystal growth can be seen within a much shorter duration. The results showed that number of the adherent crystals on MDCK renal tubular cell surfaces was significantly decreased in caffeine-treated group, in a dose-dependent manner (Fig. 3). These data indicated that caffeine could reduce COM crystal-binding capacity of MDCK renal tubular cells.

**Figure 4:**
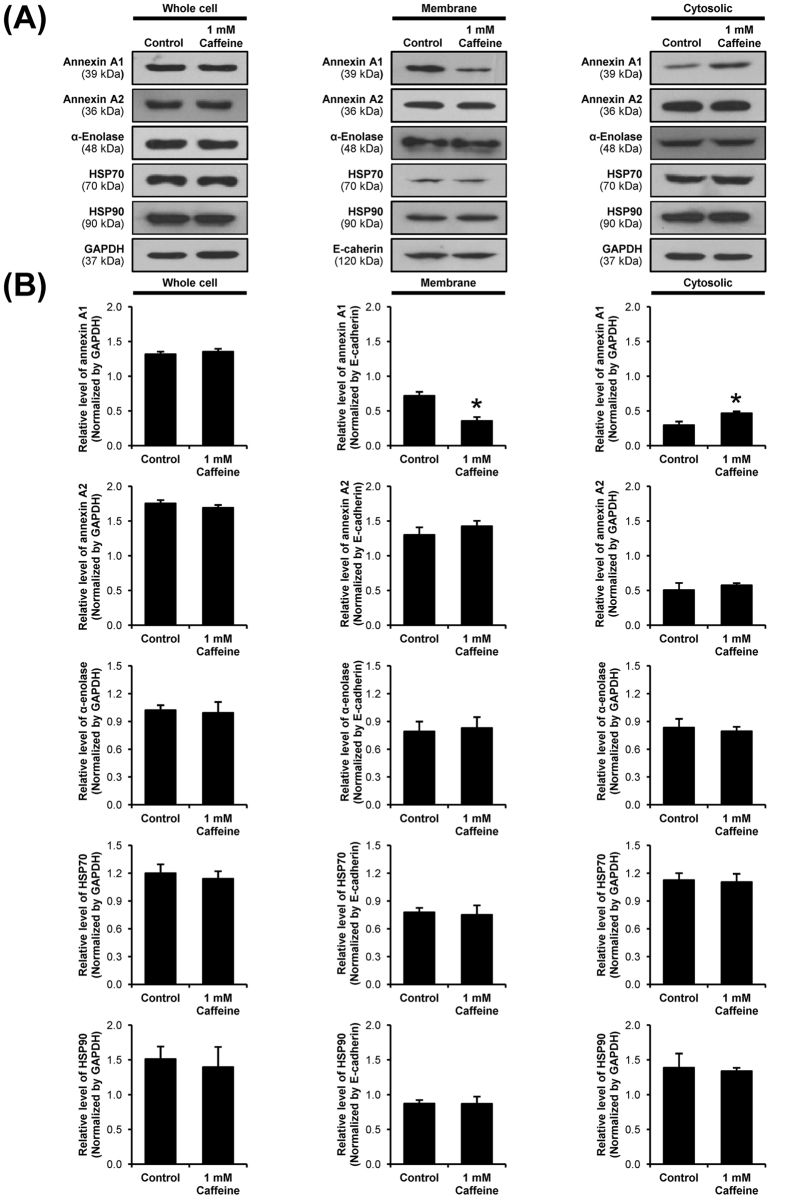
Western blot analysis of known COM crystal-binding proteins. (**A**) Proteins derived from whole cell lysate, membrane fraction, and cytosolic fraction of the cells without or with 1 mM caffeine treatment were subjected to Western blotting of annexin A1, annexin A2, α-enolase, HSP70 and HSP90. GAPDH served as the loading control for whole cell lysate and cytosolic fraction, whereas E-cadherin served as the loading control for membrane fraction. Full-length blots of these cropped images are presented in Supplementary Figure S1. (**B**) Band intensity was quantitated using ImageQuant TL software (GE healthcare). Each bar represents mean ± SEM from 3 independent experiments. *p < 0.05 vs. control.

The COM crystal-binding capacity of renal tubular epithelial cells depends largely on expression of COM crystal-binding molecules on their apical surfaces^21^,^22^. Recently, a number of COM crystal-binding proteins have been identified by a high-throughput proteomics approach^23^. We thus examined changes in expression levels of these COM crystal-binding proteins in MDCK cells after the cells were treated with 1 mM caffeine. Note that we selected this dosage as it showed modest degree of difference as compared to the higher dose (10 mM), but had obvious differences as compared to lower doses (1–100 μM). Western blot analysis of known COM crystal-binding proteins (including annexin A1, annexin A2, α-enolase, HSP70, and HSP90) were performed in whole cell lysate, membrane fraction and cytosolic fraction of MDCK cells. The band intensity data showed significant decreased level of annexin A1 in membrane fraction, whereas its cytosolic level was increased (Fig. 4). However, level of annexin A1 in whole cell lysate was unchanged, indicating that caffeine caused translocation of annexin A1 rather than decreased production/translation (Fig. 4). The data also indicated that caffeine specifically affected membrane expression of annexin A1, but did not affect expression of other COM crystal-binding proteins (Fig. 4).

**Figure 5:**
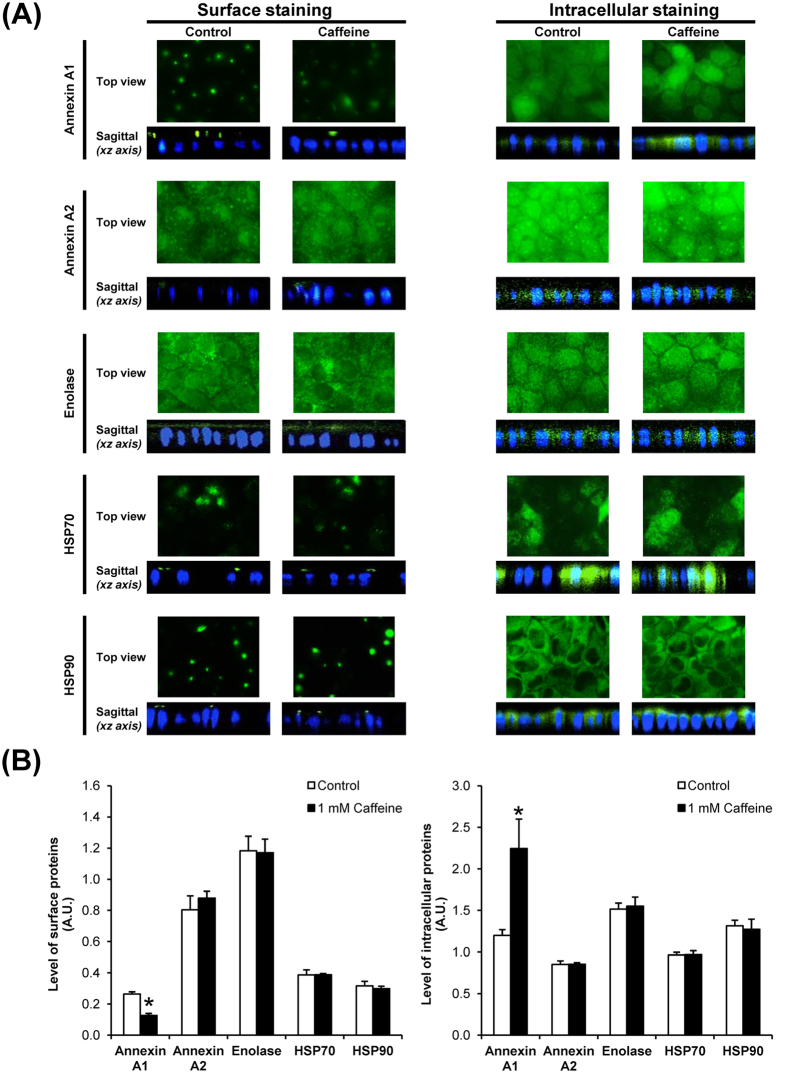
Immunofluorescence staining and laser-scanning confocal microscopy of known COM crystal-binding proteins. (**A**) The fixed cells without permeabilization were processed for apical surface staining, whereas the fixed and permeabilized cells were processed for intracellular staining of known COM crystal-binding proteins. After incubation with specific primary antibody and corresponding secondary antibody conjugated with Alexa Fluor 488, each protein was visualized (in green) using a laser-scanning confocal microscope (ECLIPSE Ti-Clsi4 Laser Unit) (Nikon). The nuclei are shown in blue. Original magnification was 630× for all panels. (**B**) The mean fluorescence intensity of each protein was analyzed using NIS-Elements D V.4.11 software (Nikon) from at least 100 individual cells in each condition. Each bar represents mean ± SEM from 3 independent experiments. Exposure time = 400 ms; A.U. = arbitrary unit; *p < 0.05 vs. control.

To further confirmed that caffeine specifically affected translocation of annexin A1 from membrane into cytoplasm, immunofluorescence staining of all these COM crystal-binding proteins followed by laser-scanning confocal microscopy was performed. The immunofluorescence data confirmed that annexin A1 expression on apical surface was significantly decreased, whereas its intracellular expression was increased (Fig. 5), whereas there were no significant changes observed for other COM crystal-binding proteins (Fig. 5). These immunofluorescence findings were consistent with the Western blotting data (Fig. 4), strengthening the specific effect of caffeine on annexin A1 expression.

**Figure 6:**
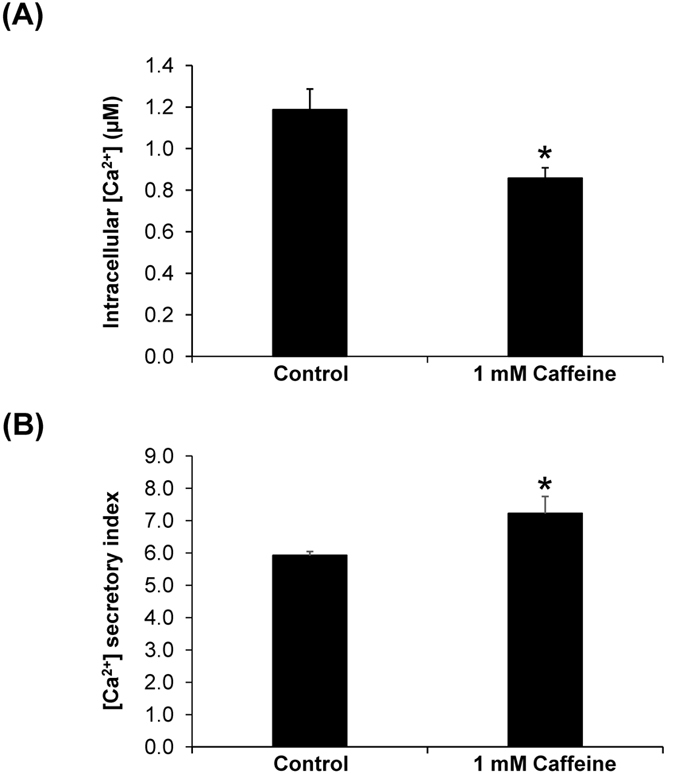
Measurement of intracellular [Ca^2+^] and [Ca^2+^] secretory index. After treatment with 1 mM caffeine for 3-h, intracellular and secretory [Ca^2+^] contents were measured using a method based on Arsenazo III-calcium reaction (see details in “Materials and Methods”). (**A**) Intracellular [Ca^2+^]; (**B**) [Ca^2+^] secretory index was calculated using ***Equation 3:*** [*Ca*^*2*+^] *secretory index* = ([*Ca*^*2*+^]_*Sample Sup*_ − [*Ca*^*2*+^]_*MEM*_)*/*[*Ca*^*2*+^]_*MEM*_ × *100*. Each bar represents mean ± SEM from 3 independent experiments. *p < 0.05 vs. control.

**Figure 7:**
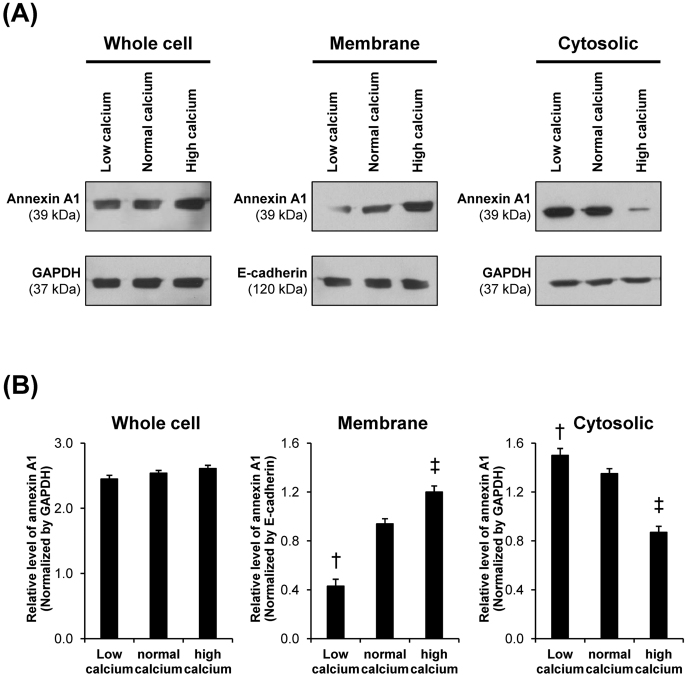
Western blotting analysis of effects of differential calcium levels on annexin A1 expression level and localization. (**A**) Proteins derived from whole cell lysate, membrane fraction, and cytosolic fraction of the cells maintained in media with differential calcium levels, including low-calcium (with 0.2 mM calcium), normal-calcium (with 1.8 mM calcium), and high-calcium (with 20 mM calcium) media (details are provided in Supplementary Table S1) for 3-h were subjected to Western blotting of annexin A1. GAPDH served as the loading control for whole cell lysate and cytosolic fraction, whereas E-cadherin served as the loading control for membrane fraction. Full-length blots of these cropped images are presented in Supplementary Figure S2. (**B**) Band intensity was quantitated using ImageQuant TL software (GE healthcare). Each bar represents mean ± SEM from 3 independent experiments. ^†^*p* < 0.05 vs. normal-calcium and high-calcium conditions; ^‡^*p* < 0.05 vs. normal-calcium and low-calcium conditions.

**Figure 8:**
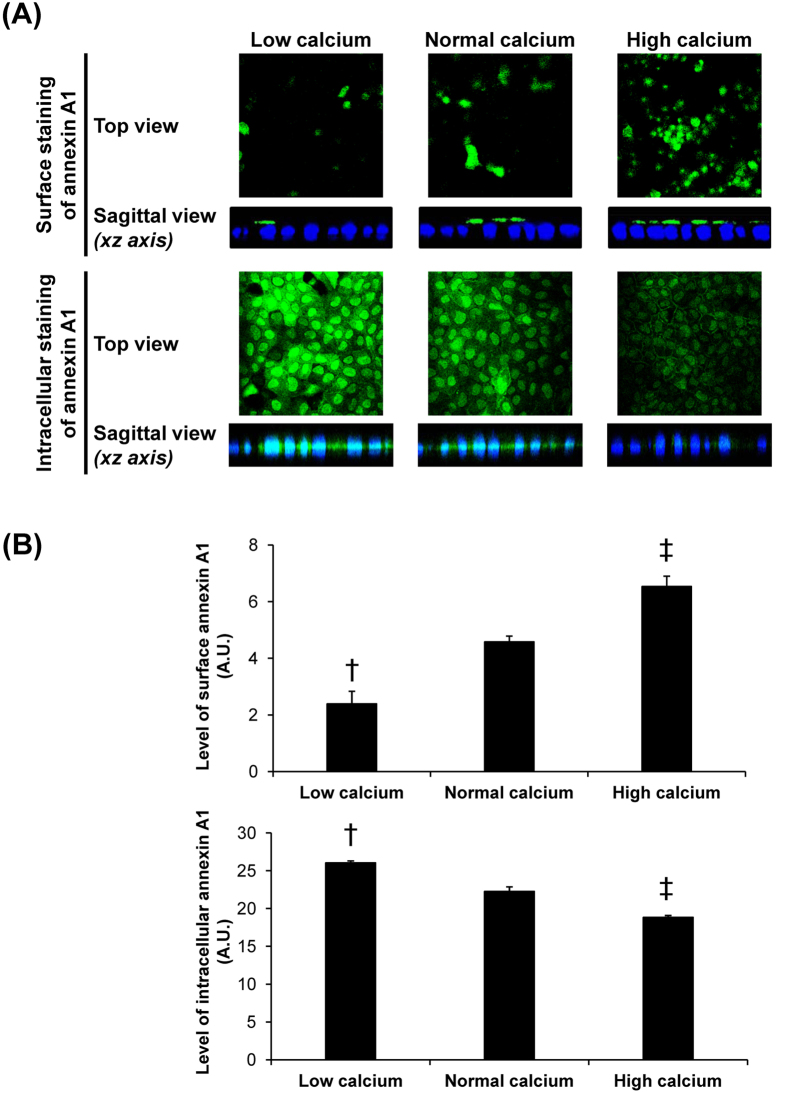
Immunofluorescence study of effects of differential calcium levels on annexin A1 expression level and localization. (**A**) The fixed cells without permeabilization were processed for apical surface staining, whereas the fixed and permeabilized cells were processed for intracellular staining of annexin A1. After incubation with mouse monoclonal anti-annexin A1 (Santa Cruz Biotechnology) and corresponding secondary antibody conjugated with Alexa Fluor 488, surface and intracellular annexin A1 was visualized (in green) using a laser-scanning confocal microscope (ECLIPSE Ti-Clsi4 Laser Unit) (Nikon). The nuclei are shown in blue. Original magnification was 630× for all panels. (**B**) The mean fluorescence intensity of annexin A1 in each condition was analyzed using NIS-Elements D V.4.11 software (Nikon) from at least 100 individual cells per condition. Each bar represents mean ± SEM from 3 independent experiments. Exposure time = 400 ms; A.U. = arbitrary unit; ^†^*p* < 0.05 vs. normal-calcium and high-calcium conditions; ^‡^*p* < 0.05 vs. normal-calcium and low-calcium conditions.

To define mechanisms underlying caffeine-induced changes in annexin A1, intracellular [Ca^2+^] and [Ca^2+^] secretory index were measured. The data showed that intracellular [Ca^2+^] was significantly decreased, whereas [Ca^2+^] secretory index was significantly increased by caffeine treatment (Fig. 6). These data were consistent with those reported in a previous study, which demonstrated calcium release from intracellular calcium storage by caffeine treatment^24^. To further confirm the effects of differential calcium levels on annexin A1 translocation in renal tubular cells, we examined expression level and localization of annexin A1 in polarized MDCK cells maintained in low-calcium (0.2 mM), normal-calcium (1.8 mM) and high-calcium (20 mM) conditions. Western blotting of annexin A1 in whole cell, apical membrane and cytosolic fractions revealed no significant difference in total level of annexin A1 (in whole cell lysate) among the three conditions (Fig. 7). However, low-calcium decreased apical membrane but increased cytosolic level of annexin A1, whereas high-calcium caused the opposite results (Fig. 7). Immunofluorescence study of annexin A1 on apical membrane surface (without permeabilization) and intracellularly (with permeabilization) confirmed the Western blotting data (Fig. 8).

Caffeine is the most abundant methylxanthine compound found in several common beverages, including coffee, cocoa, tea, soft drinks, and energy drinks. After consumption, caffeine is rapidly and completely absorbed by gastrointestinal tract and further distributes to various tissues, including the kidney. The absorbed caffeine is then metabolized, whereas 0.5–3.0% of its absorbed compartment remains unchanged and excreted into the urine^25^,^26^,^27^. In the present study, various doses of caffeine (from 1 μM to 10 mM) were used to study the protective mechanisms of caffeine at early phases of COM kidney stone formation, including crystallization, crystal growth, and crystal-cell adhesion^28^. This range of caffeine dosage was chosen based on the urinary caffeine concentrations measured after 3-h of caffeinated beverage ingestion^29^,^30^,^31^, as well as concentrations used in previous studies on effects of caffeine on the kidney^32^,^33^. Previous studies have demonstrated that caffeine increased urinary excretion of citric acid^34^, which is considered as one among several COM crystal growth inhibitors, but on the other hand enhanced urinary calcium excretion^9^,^34^,^35^,^36^. While increased urinary citrate may inhibit COM crystallization and crystal growth, the increased urinary calcium excretion is a counterbalance. Therefore, it is unlikely that caffeine reduces the risk of kidney stone disease by this mechanism. The findings from our present study confirm that caffeine does not affect overall crystallization and crystal growth.

Annexin A1 is in a family of calcium- and phospholipid-binding proteins that play significant roles in controlling intracellular calcium releasing as well as calcium-dependent signal transduction pathways. In physiologic condition, annexin A1 can be found in cytoplasm, plasma membrane and organelle membranes^37^. During low-calcium state, it is expressed in a soluble form found mainly in the cytoplasm^37^. Our previous study using high-throughput proteomics approach has shown that high-calcium condition increased apical surface expression of annexin A1 and consequently enhanced COM crystal-binding capacity of MDCK cells^12^. In contrast, our present study found that annexin A1 was translocated from apical membrane to cytoplasmic compartment in response to caffeine treatment. As aforementioned, recent studies have demonstrated that caffeine could enhance urinary calcium excretion^9^,^34^,^35^,^36^. It was thus plausible that caffeine might cause urinary loss of calcium leading to calcium deprivation state inside the cells and thus caused a reduction of membrane-associated form of annexin A1 and its translocation into cytoplasm. These hypothesis was confirmed by our data, which showed that [Ca^2+^] secretory index was increased from the caffeine-treated renal tubular epithelial cells resulting to reduction of intracellular [Ca^2+^] (Fig. 6). And calcium-deprivation state (represented by low-calcium condition) resulted to reduced expression of apical membrane annexin A1, whereas intracellular annexin A1 was increased (Figs 7 and 8). Taken together, these data indicated that caffeine increased calcium secretion/excretion and caused deprivation of calcium inside the cells, resulting to translocation of annexin A1 from apical surface into cytoplasm.

Although annexins A1 and A2 are the proteins within the same family that share the calcium ion-binding properties, our previous and present studies have shown that their responses to high-calcium^12^ and caffeine totally differed – only annexin A1 that was affected by high-calcium^12^ and caffeine. This difference between the two annexins might be due to their specific or unique functions^38^. For example, annexin A2 can bind to the membranes in a calcium-independent manner^39^, and thus was not affected by caffeine.

In summary, our present study has demonstrated that caffeine did not affect total COM crystallization product (i.e., crystal mass) and crystal growth. However, it could reduce intracellular calcium storage by increasing calcium secretion/excretion, resulting to translocation of annexin A1 from apical membrane into cytoplasm and finally the decrease of COM crystal-binding capacity of renal tubular epithelial cells. These *in vitro* data may, at least in part, explain the association of caffeinated beverage consumption with lower risk of kidney stone formation that may lead to the development of protective strategies to prevent kidney stone disease and its recurrence.

## Materials and Methods

### COM crystallization assay

COM crystallization assay was performed according to a protocol established previously^18^,^40^. Briefly, 0.5 ml of 10 mM calcium chloride (Sigma-Aldrich; St. Louis, MO) in basic buffer containing 10 mM Tris and 90 mM NaCl (VWR; Leuven, Belgium) was added into 24-well polystyrene plate (Corning Inc.; Corning, NY) in the absence or presence of purified caffeine (Sigma) with various concentrations of 1 μM, 10 μM, 100 μM, 1 mM, and 10 mM. For the control, an equal volume of the basic buffer was added instead of caffeine. Thereafter, 0.5 ml of 1 mM sodium oxalate (Sigma-Aldrich) in basic buffer was added into each well of the mixture. After 60-min incubation at room temperature (RT) (set at 25 °C), the crystal images were taken using an inverted light microscope (Nikon eclipse Ti; Nikon; Tokyo, Japan). Crystal size was then analyzed using crystal area measured by NIS-element D V.4.11 software (Nikon) from at least 100 individual crystals in each well. Crystal number in each well was counted from 15 high-power fields (HPFs). Crystal mass was calculated using the formula:


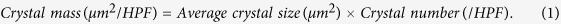


### COM crystal growth assay

COM crystals were prepared in 24-well polystyrene plate by mixing 10 mM calcium chloride with 1 mM sodium oxalate in basic buffer to make their final concentrations of 5 mM and 0.5 mM, respectively. After 60-min incubation at RT, caffeine with various doses (1 μM, 10 μM, 100 μM, 1 mM, and 10 mM) was added to the crystal mixture and the crystals were immediately imaged (set as T_0_) under an inverted light microscope (Nikon eclipse Ti). After further 60-min incubation at RT, crystal images were then again captured (set as T_60_) under an inverted light microscope (Nikon eclipse Ti). The crystal sizes at T_0_ and T_60_ were then analyzed from at least 100 individual crystals per well using NIS-element D V.4.11 software (Nikon) and crystal growth was calculated from Δ crystal area using the formula:


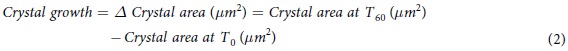


### Cell cultivation

Renal tubular cells (MDCK cell line) were grown in a complete medium containing minimum essential medium (MEM) (Gibco; Life Technologies Corporation; Grand Island, NY) supplemented with 10% fetal bovine serum (FBS) (Gibco), 2 mM L-glutamine and 1.2% penicillin/streptomycin (Sigma-Aldrich). The cells were maintained in a humidified incubator (Thermo Fisher Scientific; Marietta, OH) with 5% CO_2_ at 37 °C. To develop polarization, the cells at a density of 7.5 × 10^4 ^cells/ml were seeded and grown on prewetted collagen-coated permeable polycarbonate membrane insert in Transwells (0.4 μm pore size) (Corstar; Cambridge, MA). The complete medium was refreshed every other day for four days or until they became fully polarized epithelial cells.

To examine effects of differential calcium levels on annexin A1 expression level and localization, three types of media, including low-calcium (with 0.2 mM calcium), normal-calcium (with 1.8 mM calcium), and high-calcium (with 20 mM calcium) media were prepared in-house. All compositions including other inorganic salts, amino acids and vitamins are detailed in Supplementary Table S1. The polarized cells were maintained in these in-house media with differential calcium levels for 3-h prior to other experiments.

### Cell-crystal adhesion assay

COM crystals were prepared by mixing 10 mM calcium chloride with 1 mM sodium oxalate in basic buffer to make their final concentrations of 5 mM and 0.5 mM, respectively The mixture was incubated at RT overnight and then centrifuged at 3,000 rpm for 5 min. COM crystal pellets were collected, resuspended in methanol and then centrifuged at 3,000 rpm for 5 min. Methanol was removed and COM crystals were air-dried and then de-contaminated by UV radiation for 30 min before use. To ensure the consistency of the assay among different conditions in each replicate, MDCK cells (approximately 5 × 10^5^ cells) were seeded into each well of the same 6-well cell culture plate (Corning Inc.) and maintained in a complete medium for 24 h. The cells were then treated with caffeine (1 μM, 10 μM, 100 μM, 1 mM or 10 mM) for 3-h, whereas the cells maintained in medium without caffeine served as the control. The culture medium was gently removed and the cells were rinsed with plain MEM. The cells were further incubated with COM crystals (at a concentration of 100 μg crystals in 1 ml culture medium) for 30 min. The cells were then vigorously washed with plain MEM 5 times to remove non-adhered COM crystals. The adhered crystals were observed, imaged and counted under a phase-contrast microscope (Nikon) from at least 15 HPFs in each well.

### Subcellular fractionation

Approximately 5 × 10^5^ MDCK cells were grown in 6-well cell culture plate for 24 h before treatment with 1 mM caffeine or with media with differential calcium levels, including low-calcium (with 0.2 mM calcium), normal-calcium (with 1.8 mM calcium), and high-calcium (with 20 mM calcium) media (details are provided in Supplementary Table S1) for 3-h. Thereafter, the cells were rinsed with ice-cold membrane preserving buffer (PBS containing 1 mM MgCl_2_ and 0.1 mM CaCl_2_) and then incubated with cytosolic extraction buffer (10 mM PIPES pH 6.8, 0.02% digitonin, 0.3 mM sucrose, 15 mM NaCl, and 0.5 mM EDTA)^41^ at 4 °C for 10 min. The supernatant containing cytosolic proteins was then collected as “cytosolic fraction”. The remaining cells were further washed three times with ice-cold membrane preserving buffer and then extracted by Laemmli’s buffer to collect the “membrane fraction”. The “whole cell lysate” was prepared by extracting whole cells using Laemmli’s buffer. Protein concentration in each sample was determined by Bradford’s method using Bio-Rad Protein Assay (Bio-Rad Laboratories; Hercules, CA).

### Western blotting of known COM crystal-binding proteins

Equal amount (30 μg) of proteins derived from whole cell lysate, cytosolic fraction, and membrane fraction as aforementioned were resolved by 12% SDS-PAGE. The resolved proteins were then transferred onto a nitrocellulose membrane (Whatman; Buckinghamshire, UK) and non-specific bindings were blocked with 5% skim milk (Sigma-Aldrich)/PBS for 30 min. The membranes were then incubated with mouse monoclonal anti-annexin A1, goat polyclonal anti-annexin A2, rabbit polyclonal anti-α-enolase, mouse monoclonal anti-HSP70, mouse monoclonal anti-HSP90, mouse monoclonal anti-GAPDH, or rat polyclonal anti-E-cadherin antibody (all the primary antibodies were purchased from Santa Cruz Biotechnology; Santa Cruz, CA; and diluted 1:1,000 in 1% skim milk/PBS) at 4 °C overnight. The membrane was washed three times with PBS followed by incubation with corresponding secondary antibody conjugated with horseradish peroxidase (HRP) (Dako; Glostrup, Denmark) (1:2,000 in 1% skim milk/PBS) at RT for 1 h. After other three washes, the immunoreactive bands were developed using SuperSignal West Pico chemiluminescence substrate (Pierce Biotechnology; Rockford, IL) and then visualized by autoradiogram. The band intensity was then analyzed using ImageQuant TL software (GE healthcare; Uppsala, Sweden).

### Immunofluorescence staining of known COM crystal-binding proteins followed by laser-scanning confocal microscopy

MDCK cells, approximately 2.5 × 10^5^ cells, were grown on coverslip for 24 h and then treated with 1 mM caffeine or with media with differential calcium levels, including low-calcium (with 0.2 mM calcium), normal-calcium (with 1.8 mM calcium), and high-calcium (with 20 mM calcium) media (details are provided in Supplementary Table S1) for 3-h. After washing with membrane preserving buffer, the cells were fixed with 4% paraformaldehyde at RT for 10 min. For apical surface staining, the fixed cells were further incubated with primary antibody without permeabilization. For intracellular staining, the fixed cells were permeabilized with 0.1% TritonX-100 at RT for 10 min prior to incubation with primary antibody. For both apical surface and intracellular stainings, the cells were incubated with mouse monoclonal anti-annexin A1, goat polyclonal anti-annexin A2, rabbit polyclonal anti-α-enolase, mouse monoclonal anti-HSP70, or mouse monoclonal anti-HSP90 antibody (all were from Santa Cruz Biotechnology and diluted 1:50 in 1%BSA/PBS) at 37 °C for 1 h. The cells were then washed three times with ice-cold membrane preserving buffer before further incubated at 37 °C for 1 h with corresponding secondary antibody conjugated with Alexa Fluor 488 (Molecular Probes; Burlington, Canada) (1:2,000 in 1%BSA/PBS) mixed with 0.1 μg/ml Hoechst dye (Invitrogen; Paisley, UK) to stain nuclei. Thereafter, the coverslip was mounted with 50% glycerol/PBS. Apical surface and intracellular expression of known COM crystal-binding proteins was evaluated by a laser-scanning confocal microscope (ECLIPSE Ti-Clsi4 Laser Unit) (Nikon; Tokyo, Japan). The mean fluorescence intensity of each protein was analyzed using NIS-Elements D V.4.11 software (Nikon) from at least 100 individual cells in each condition.

### Measurement of intracellular [Ca^2+^] and [Ca^2+^] secretory index

MDCK cells, approximately 5 × 10^5^ cells, were grown in each well of 6-well plate for 24 h. After 3-h treatment with caffeine as for other aforementioned assays, the supernatant was collected for measurement of [Ca^2+^] secretory index, whereas the cells were washed with PBS before lysis in deionized water (hypoosmotic burst). After lysis, cellular debris was removed by centrifugation at 10,000 *g* and 4 °C for 5 min and the cell lysate was subjected to intracellular [Ca^2+^] measurement. Intracellular calcium and calcium secretion/excretion were measured using a method based on Arsenazo III-calcium reaction as previously described^42^. Briefly, the sample (1.5 μl each) was transferred into each well of 96-well polystyrene microplate followed by an addition of 100 μl Arsenazo III reagent (BioSystems S.A.; Barcelona, Spain). After 5-min incubation at RT, [Ca^2+^] in each sample well was measured at λ 620 nm using a microplate reader (EZ Read 400) (Biochrom; Holliston, MA). The [Ca^2+^] secretory index was calculated by the following formula:


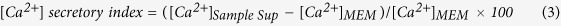


where [Ca^2+^]_Sample Sup_ was the calcium concentration measured from each well of culture supernatant, whereas [Ca^2+^]_MEM_ was the basal concentration of MEM culture medium prior to experiment (which was approximately 1.8 mM).

### Statistical analysis

All quantitative data are reported as mean ± SEM from 3 independent experiments. The mean differences between two groups were analyzed by unpaired *t-*test, whereas multiple comparisons among several groups were analyzed by one-way ANOVA with Tukey’s post-hoc test. Statistically significant threshold was set at *p* < 0.05.

## Additional Information

**How to cite this article**: Peerapen, P. and Thongboonkerd, V. Caffeine prevents kidney stone formation by translocation of apical surface annexin A1 crystal-binding protein into cytoplasm: *In vitro* evidence. *Sci. Rep.* **6**, 38536; doi: 10.1038/srep38536 (2016).

**Publisher’s note:** Springer Nature remains neutral with regard to jurisdictional claims in published maps and institutional affiliations.

## Supplementary Material

Supplementary Tables and Figures
