# Supplementary material for: Caffeine prevents kidney stone formation by translocation of apical surface annexin A1 crystal-binding protein into cytoplasm: In vitro evidence
Source: Sci Rep. 2016 Dec 7;6:38536. doi: 10.1038/srep38536 (PMC5141452; doi:10.1038/srep38536)

Caffeine prevents kidney stone formation by translocation of apical surface annexin A1 crystal-binding protein into cytoplasm: *In vitro* evidence

Paleerath Peerapen and Visith Thongboonkerd (correspondence to: vthongbo@yahoo.com)*

Supplementary Table S1: Compositions of in-house media with differential calcium levels.

Components	Concentration (g/L)		
	**Low Ca^{2+} ($[Ca^{2+}]$ = 0.2 mM)**	**Normal Ca^{2+} ($[Ca^{2+}]$ = 1.8 mM)**	**High Ca^{2+} ($[Ca^{2+}]$ = 20 mM)**
**Inorganic salts**			
Calcium chloride dihydrate	0.0294	0.2646	2.9402
Magnesium sulfate	0.2002	0.2002	0.2002
Potassium chloride	0.4000	0.4000	0.4000
Sodium bicarbonate	1.5000	1.5000	1.5000
Sodium chloride	6.8000	6.8000	6.8000
Sodium Phosphate monobasic	0.1400	0.1400	0.1400
**Amino acids**			
Essential amino acid (M-5550, Sigma-Aldrich)			
L-Arginine.HCl	0.1266	0.1266	0.1266
L-Cystine.2HCl	0.0313	0.0313	0.0313
L-Glutamine	0.2920	0.2920	0.2920
L-Histidine.HCl.H_2O	0.0420	0.0420	0.0420
L-Isoleucine	0.0525	0.0525	0.0525
L-Leucine	0.0524	0.0524	0.0524
L-Lysine.HCl	0.0725	0.0725	0.0725
L-Methionine	0.0151	0.0151	0.0151
L-Phenylalanine	0.0330	0.0330	0.0330
L-threonine	0.0476	0.0476	0.0476
L-Tryptophan	0.0102	0.0102	0.0102
L-Tyrosine	0.0560	0.0560	0.0560
L-Valine	0.0468	0.0468	0.0468
Non-essential amino acids (M-7145, Sigma-Aldrich)			
L-Alanine	0.0089	0.0089	0.0089
L-Asparagine.H_2O	0.0150	0.0150	0.0150
L-Aspartic acid	0.0133	0.0133	0.0133
L-Glutamic acid	0.0147	0.0147	0.0147
L-Glycine	0.0075	0.0075	0.0075
L-Proline	0.0115	0.0115	0.0115
L-Serine	0.0105	0.0105	0.0105
**Vitamins** (M-6895, Sigma-Aldrichl)			
D-Ca Pantothenic acid	0.0010	0.0010	0.0010
Choline chloride	0.0010	0.0010	0.0010
Folic acid	0.0010	0.0010	0.0010
i-Inositol	0.0020	0.0020	0.0020
Niacinamide	0.0010	0.0010	0.0010
Pyridoxal.HCl	0.0010	0.0010	0.0010
Riboflavin	0.0001	0.0001	0.0001
Thiamine.HCl	0.0010	0.0010	0.0010
**Other components**			
D-Glucose	1.0000	1.0000	1.0000
Phenol red	0.0100	0.0100	0.0100

Supplementary Figure S1: Full-length blots of known COM crystal-binding proteins (shown as cropped images in **Figure 4**).

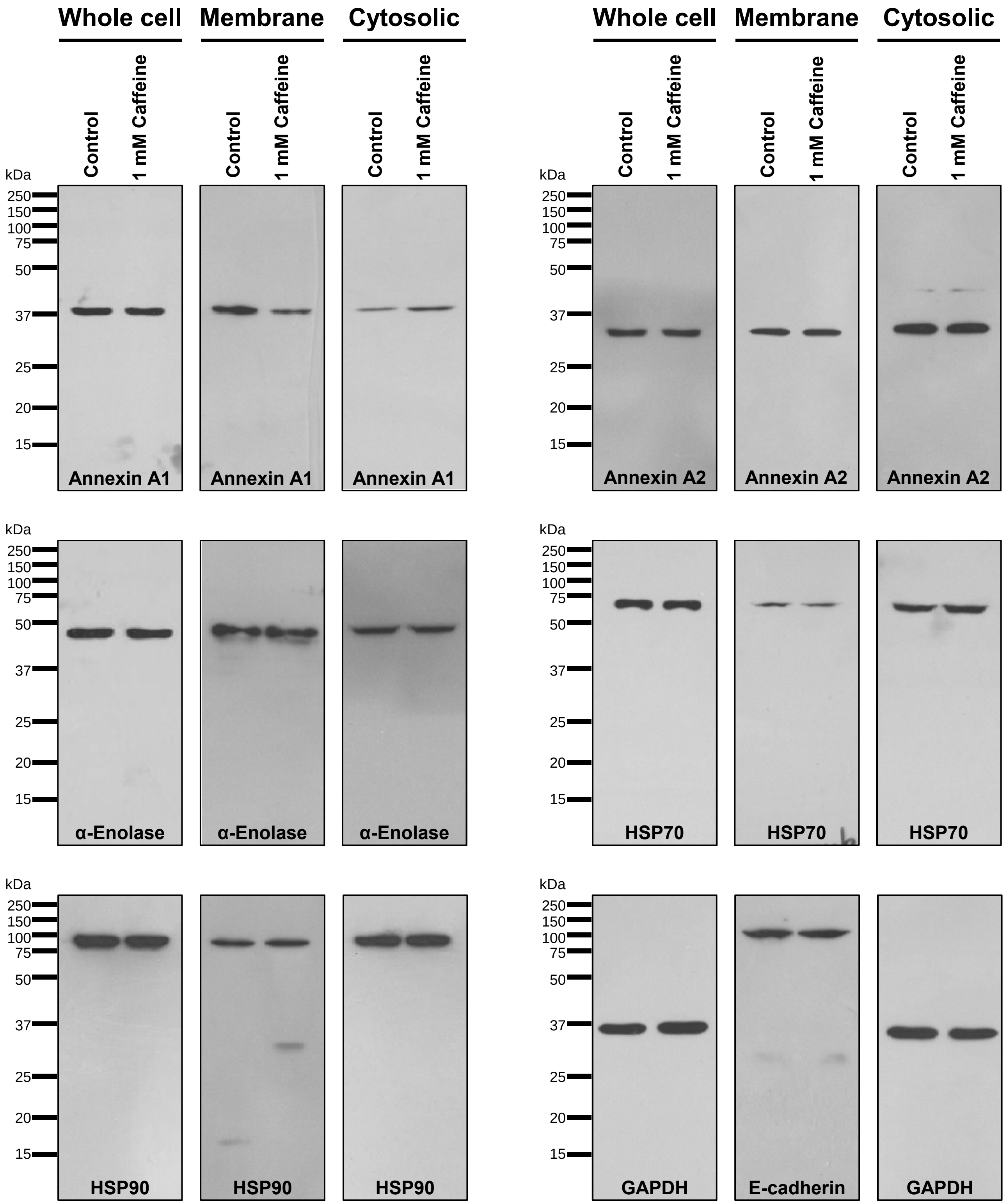

Supplementary Figure S2: Full-length blots of Annexin A1 in whole cell, membrane, and cytosolic fractions (shown as cropped images in **Figure 7**).

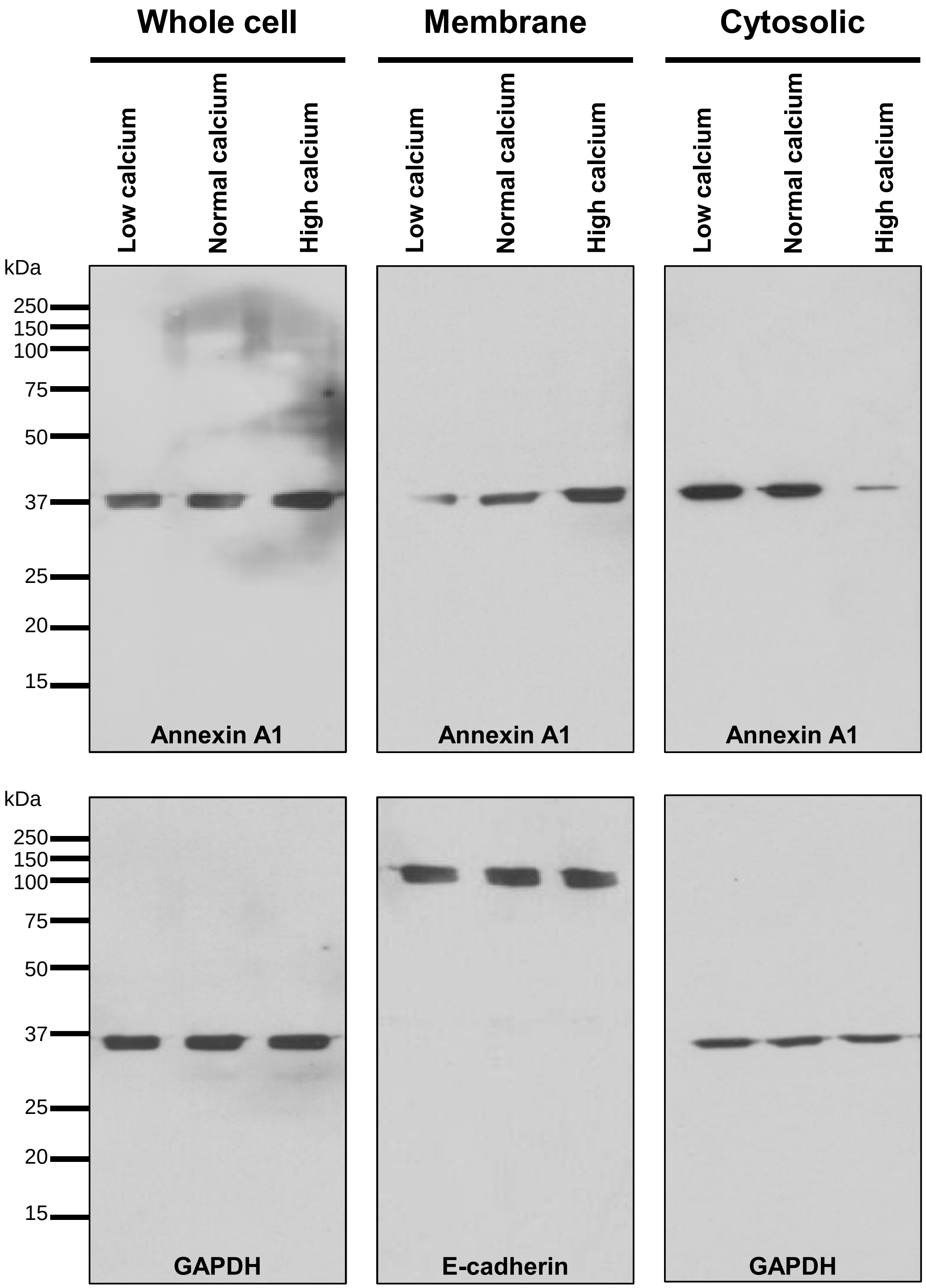

Supplement: Supplementary Tables and Figures [file srep38536-s1.pdf]
